# New Benzotriazole and Benzodithiophene-Based Conjugated Terpolymer Bearing a Fluorescein Derivative as Side-Group: In-Ternal Förster Resonance Energy Transfer to Improve Organic Solar Cells

**DOI:** 10.3390/ijms232112901

**Published:** 2022-10-26

**Authors:** Ignacio A. Jessop, Josefa Cutipa, Yasmín Perez, Cesar Saldías, Denis Fuentealba, Alain Tundidor-Camba, Claudio A. Terraza, María B. Camarada, Felipe A. Angel

**Affiliations:** 1 Organic and Polymeric Materials Research Laboratory, Facultad de Ciencias, Universidad de Tarapacá, P.O. Box 7-D, Arica 1000007, Chile; 2 Departamento de Química Física, Escuela de Química, Facultad de Química y de Farmacia, Pontificia Universidad Católica de Chile, Santiago 7820436, Chile; 3 Research Laboratory for Organic Polymers (RLOP), Faculty of Chemistry and of Pharmacy, Pontificia Universidad Católica de Chile, P.O. Box 306, Post 22, Santiago 7820436, Chile; 4 UC Energy Research Center, Pontificia Universidad Católica de Chile, Santiago 7820436, Chile; 5 Departamento de Química Inorgánica, Escuela de Química, Facultad de Química y de Farmacia, Pontificia Universidad Católica de Chile, Santiago 7820436, Chile; 6 Centro de Nanotecnología y Materiales Avanzados, CIEN-UC, Pontificia Universidad Católica de Chile, Santiago 7820436, Chile

**Keywords:** π-Conjugated polymers, benzotriazole, benzodithiophene, fluorescein, FRET, organic solar cells

## Abstract

A new benzodithiophene and benzotriazole-based terpolymer bearing a fluorescein derivative as a side group was synthesized and studied for organic solar cell (OSC) applications. This side group was covalently bounded to the backbone through an *n*-hexyl chain to induce the intramolecular Förster Resonance Energy Transfer (FRET) process and thus improve the photovoltaic performance of the polymeric material. The polymer exhibited good solubility in common organic chlorinated solvents as well as thermal stability (TDT_10%_ > 360 °C). Photophysical measurements demonstrated the occurrence of the FRET phenomenon between the lateral group and the terpolymer. The terpolymer exhibited an absorption band centered at 501 nm, an optical bandgap of 2.02 eV, and HOMO and LUMO energy levels of −5.30 eV and −3.28 eV, respectively. A preliminary study on terpolymer-based OSC devices showed a low power-conversion efficiency (PCE) but a higher performance than devices based on an analogous polymer without the fluorescein derivative. These results mean that the design presented here is a promising strategy to improve the performance of polymers used in OSCs.

## 1. Introduction

π-Conjugated polymers (CPs) have been widely studied during the last decades due to the unique combination of optical and electronic properties, such as sunlight harvesting capability and charge carrier mobility, with those inherent to synthetic polymers, such as structural and functional versatility, solution processability, flexibility, and light weight [1,2,3]. This set of properties makes them suitable for many commercial applications, such as organic solar cells (OSCs), organic light-emitting diodes (OLEDs), organic field-effect transistors (OFET), and sensors, among others [4,5,6,7,8]. Regarding its photovoltaic applications, the CPs must have a relatively low bandgap, suitable HOMO/LUMO energy levels, broad absorption in the visible region, solubility in common organic solvents, and proper charge transport to be used as active layers in high-performance OSCs [9,10]. The power-conversion energy (PCE) of polymer-based bulk-heterojunction (BHJ) OSC devices has increased significantly in recent years, overcoming the 18% barrier [11,12,13,14]. This advance has been driven mainly by developing new high-performance materials (new donor-acceptor CPs and non-fullerene acceptors (NFAs)), different device structures (ternary and tandem solar cells), and innovation in manufacturing techniques [1,14,15]. However, challenges, such as device efficiency and stability, must be addressed to consolidate the technology in the solar market [16,17,18,19]. Regarding the improvement of the PCE, one approach is the design of new simple, low-cost, and scalable photoactive CP materials that are competitive against PM6, a benzo [1,2-*b*:4,5-*b′*]dithiophene (BDT)-based polymer that combined with NFAs has driven the growth and significant improvements in OSCs during the last few years [20,21]. The use of NFAs has brought back into the game wide-bandgap CPs, which do not require such sophisticated or complex structures to produce high PCEs [21,22,23]. Another approach may be using the Förster Resonance Energy Transfer (FRET) process between two molecules by long-range dipole–dipole coupling [24,25,26]. A large body of literature on CP-based ternary solar cells suggests that FRET improves the PCE of devices by increasing exciton generation in the photoactive material and facilitating exciton migration over long distances [27,28,29,30,31,32,33,34].

How can we take these two approaches to produce new high-performance materials and devices? BDT is a widely used electron-donor building-block due to its structural versatility and excellent optical and transport properties, capable of producing high-performance CPs [20,35,36]. Among the electron-acceptor moieties, 2*H*-benzo[*d*][1,2,3]triazole (BTz) has been extensively used for photovoltaic applications [37,38,39]. The N-H bond in BTz can efficiently react with long or branched alkyl bromides, which improves the solution processability of its CP. Furthermore, the N-H group allows connecting the CP with small molecules, increasing the versatility of the polymer. We tested this idea previously by attaching a carbazole “antenna” to poly(fluorene/thiophene-alt-benzotriazole), obtaining promising results [9]. According to the literature, polymers based on BTz and BDT moieties absorb light in the same range as fluorescein derivatives (a low-cost dye) emit fluorescence [40,41,42,43,44,45,46]. These features mean that fluorescein derivatives can be used as a FRET donor while the poly(BDT-BTz) may act as a FRET acceptor. 

**Figure 1:**
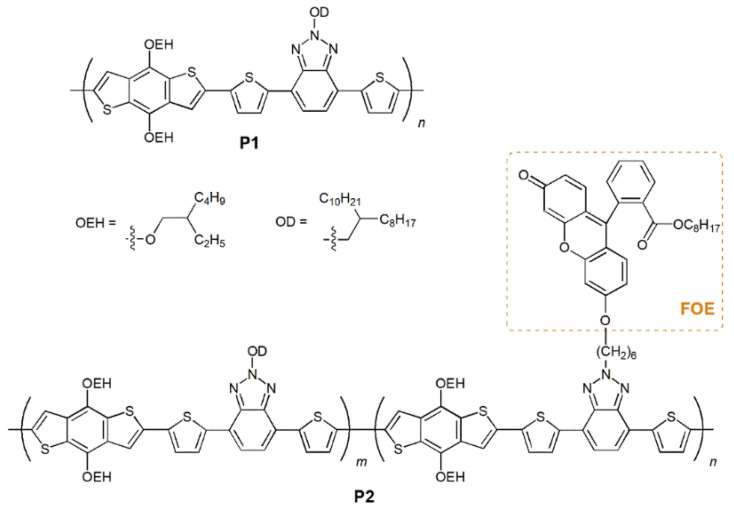
Schematic representation of the structure of P1 and P2.

Considering all these concepts, herein we report the synthesis of a new π-conjugated polymer based on BTz and BDT with a fluorescein derivative (FOE) covalently attached to its backbone (Figure 1). The emission range and significant fluorescence of FOE can promote FRET and affect the properties of the CP. A polymer without FOE was also synthesized for comparison. Both materials were synthesized via Stille polycondensation [47] and were characterized by thermal, electrochemical, photophysical and photovoltaic measurements. The results showed that promoting FRET between the side-group and a wide-bandgap polymer has excellent potential for future development.

## 2. Results and Discussion

### 2.1. Synthesis of Polymers P1 and P2

The synthetic routes for the precursors, monomers and polymers are available in the electronic supplementary information (ESI; Schemes S1 and S2). A commercially available electron-donor building-block BDT (M1) was employed, while BTz-based electron-acceptor monomers M2 and M3 units were synthesized according to previously reported procedures. These BTz-based monomers were selected due to their easily functionalizable N-H groups. M3 was prepared via S_N_2 reaction between 2-(6-bromohexyl)-4,7-bis(5-bromothiophen-2-yl)-2*H*-benzo[*d*][1,2,3]triazole and FOE (88%). FOE was obtained by conversion of the acid group on fluorescein to an ester group by acid-catalyzed Fischer esterification in 1-octanol (89 %). The octyl side chain was added to fluorescein to increase the solubility of M3 in organic solvents.

The syntheses of P1 and P2 were carried out by Stille polycondensation using Pd_2_(dba)_3_/P(*o*-tol)_3_ as a catalytic system at 110 °C in toluene (Scheme S2). Continuous Soxhlet extractions purified the polymers with methanol, *n*-hexane, and chloroform. P1 and P2 were recovered from the chloroform fraction and precipitated into methanol. After synthesizing P1 (85% yield) from M1 and M2, we focused on polymerizing M1 with M3. After several attempts, a low yield of the copolymer was obtained (<5% yield in chloroform-soluble fraction). Precipitation of the copolymer was observed during the first 20 min of the reaction, attributed to the formation of high-molecular weight polymeric chains. This feature was consistent with the high percentage of insoluble material recovered from the Soxhlet cartridge (>90%). Apparently, the structure and amount of alkyl side chains per repeating unit were insufficient to keep the polymer in solution during the growth stage. Therefore, we prepared a terpolymer by reacting M1, M2 and M3 introducing octyldodecyl side chains along the backbone to improve the solubility of the final material. After 0.5 h of reaction, terpolymer P2 was obtained in 69% yield (Figures S1 and S2). The number-average molecular weights recorded by SEC for P1 and P2 were 57 kg/mol and 42 kg/mol, with polydispersity indexes of 1.45 and 2.42, respectively. These values indicate that relatively long polymers were obtained despite short polymerization times. The polymers exhibited high thermal stability at 10% weight-loss (P1 = 364 °C and P2 = 379 °C). Besides this, P1 showed a T_g_ at 244 °C, while no thermal transitions were observed for P2 in the same thermal window (Figure S3a,b).

### 2.2. Photophysical and Electrochemical Characterization

**Figure 2:**
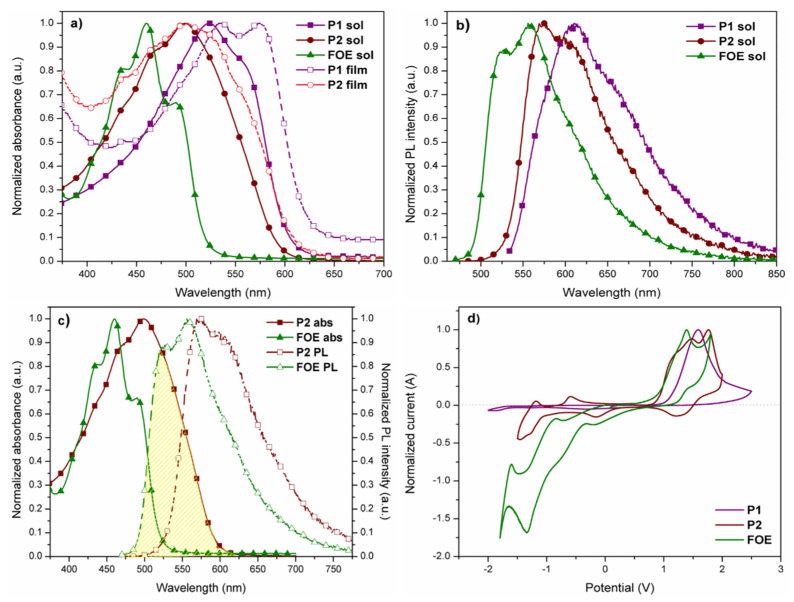
(**a**) Normalized UV-vis spectra of P1, P2, and FOE in dilute CHCl_3_ solutions and solid state. (**b**) Normalized fluorescence spectra of polymers and the side group in dilute solutions of CHCl_3_. (**c**) P2 absorption overlapping with FOE emission. (**d**) Cyclic voltammograms (second scan) of polymers and FOE drop cast on platinum disk. Scan rate = 100 m·Vs^−1^.

UV-vis absorption spectra of P1, P2, and FOE in chloroform dilute solutions and in thin-films are shown in Figure 2a, while the data are summarized in Table 1. Both polymers exhibited absorption bands in the visible region (524 nm for P1 and 499 nm for P2) due to the intramolecular charge transfer (ITC) between donor and acceptor monomers [48]. FOE absorption band (centered at 460 nm) is blue-shifted relative to polymers, causing the P2 band to broaden. The absorption band of P1 is red shifted compared to P2 in solution and solid state, which could be attributed to the difference in molecular weights between both polymers (P1 > P2). A vibrionic transition was observed for both polymers in solid-state, more clearly distinguishable in P1, indicating stronger aggregation and enhanced π-π stacking interactions [43,45,49,50,51]. The more defined peak for P1 could also indicate its higher molecular weight. The polymers showed bandgap values of around 2.0 eV in solution and thin film (Table 1).

**Figure 3:**
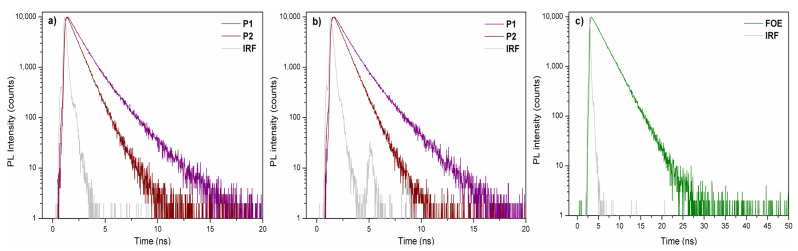
Time-resolved fluorescence spectra of P1 and P2 excited at (**a**) 458 nm and (**b**) 506 nm. (**c**) FOE spectra excited at 458 nm. IRF: Instrument Response Function.

Figure 2b shows the fluorescence spectra of P1 and P2 in chloroform dilute solutions. The maximum fluorescence peaks were observed at 610 nm (P1), 575 nm (P2), and 556 nm (FOE), following the same red-shift trend observed in UV-vis spectra, namely, P1 > P2 > FOE. As seen in Figure 2c, the FOE emission partially overlaps with the P2 absorption, making them good FRET pairs. The FOE fluorescence was expected to be quenched when the P2 solution was excited at 556 nm. However, this was not observed; therefore, time-resolved fluorescence analysis had to be performed to prove the FRET occurrence (Figure 3a–c). Table 2 shows that fluorescence lifetime (τ) of FOE was 2.64 ns. This lifetime is significantly shortened when FOE is attached to the P2 backbone, decreasing to about 1.8 ns. Conversely, much shorter fluorescence lifetimes are observed for P1. These results indicate a relatively efficient energy transfer process from FOE to the terpolymer. The energy transfer efficiency can be estimated from the lifetimes (ET = 1 − τDA·τD^−1^) [30] to be about 33%, probably because the overlap between the emission of the donor and the absorption of the acceptor is not optimal, as seen in Figure 2c.

**Figure 4:**
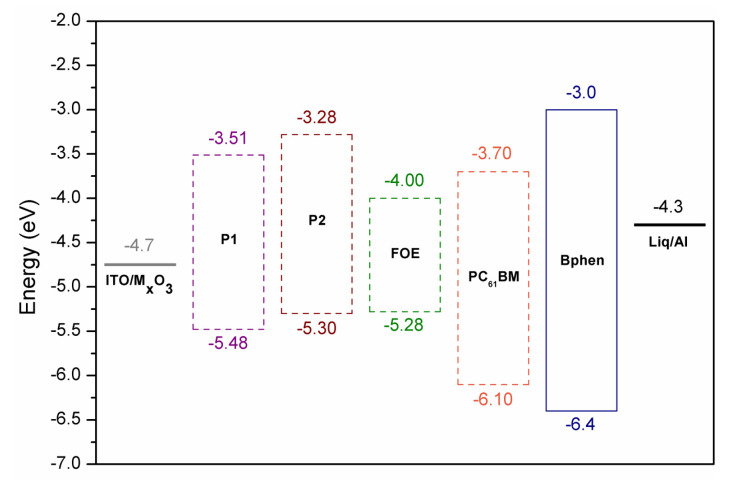
Device structure and HOMO and LUMO energy levels of P1, P2 and FOE.

The HOMO and LUMO energy levels of P1, P2, and FOE were estimated by cyclic voltammetry measurements (Figure 2d). P1 exhibited a non-reversible oxidation peak, while P2 had a quasi-reversible oxidation peak. Since the P1 voltamperogram did not show a prominent reduction peak as the oxidation peak, its LUMO energy level was calculated by the difference between its HOMO and $E_{g}^{\mathrm{opt}}$ (Table 1). $E_{\mathrm{on}}^{\mathrm{ox}}$ and $E_{\mathrm{on}}^{\mathrm{red}}$ of P2 differ from P1, since the electrochemical response of FOE hampered their estimation. The HOMO and LUMO energy values obtained for P1 and P2 are within the range reported for similar BDT- and BTz-based copolymers [43,44,46,52]. Unlike P1, P2 had a higher LUMO energy value than the LUMO of PC_61_BM, high enough (>0.3 eV) to ensure exciton dissociation (Figure 4) [53]. It is worth mentioning that the difference between the HOMOs of P2 and FOE was only 0.02 eV, which could give rise to hole traps, affecting the performance of its photovoltaic devices.

### 2.3. Photovoltaic Characterization

**Figure 5:**
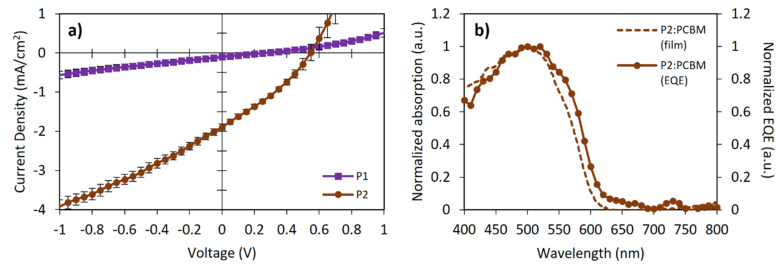
(**a**) Current density-voltage (JV) characteristics and (**b**) external quantum efficiency (EQE) for bulk-heterojunction OSC devices.

OSC devices were fabricated using the following architecture to explore the potential of FRET to improve the photovoltaic performance: ITO|MxO_3_ (8 nm)|polymer:PC_61_BM (1:1)|Bphen (8 nm)|Liq (1 nm)|Al (100 nm). Figure 5a shows the J-V curves of both polymers, while the photovoltaic parameters are summarized in Table 3. The active layer was spin-coated from chlorobenzene solutions without subsequent thermal annealing. The active layer thickness was about 55–65 nm. External quantum efficiency (EQE) measurements for the P2-based device showed that the short-circuit current density (J_sc_) value is consistent with the information obtained from the J-V curves (Figure 5b), correlating with the absorption spectra of the photoactive blend.

According to Schaber’s model [53], the open-circuit voltage (V_oc_) can be estimated from the following equation:

$$\;V_{\mathrm{oc}}=\frac{1}{e}\;\left(\left|E^{\mathrm{donor}}\mathrm{HOMO}\right|-\left|E^{\mathrm{acceptor}}\mathrm{LUMO}\right|\right)-0.3$$

where e is the electron charge and 0.3 is an empirical factor for effective exciton dissociation. From this equation, the expected V_oc_ values for P1 and P2 should have been 1.48 eV and 1.30 eV, respectively. The obtained V_oc_ values differed from those in 1.22 eV (P1) and 0.75 eV (P2). These V_oc_ losses suggested significant exciton recombination. The low J_sc_ and V_oc_ values could be attributed to the un-optimized nanomorphology of the blends and the hole traps originated between the P2 and FOE, reflected in the low fill-factor (FF) values, in addition to the fact that almost no photocurrent and spectral response were observed for P1 devices. The sum of these low parameters and the low hole mobility (Table 3) yielded poor PCEs (even despite adding 3% DIO to the blend), lower relative to those expected for poly(BDT-alt-BTz):fullerene-based devices (1.5–2.0%) [43,45,52]. Nevertheless, this preliminary study showed that all photovoltaic parameters of P2 were better than those of P1, particularly the V_oc_ and J_sc_ values. In this sense, incorporating FOE units along the BDT and BTz-based polymers effectively improved the photovoltaic response of the CP.

## 3. Materials and Methods

### 3.1. Materials

All chemical reagents and solvents were purchased from Sigma-Aldrich (Milwaukee, WI, USA) and Merck (Darmstadt, Germany) and used directly without further purification unless otherwise noted. *N*-Bromosuccinimide (NBS) was recrystallized twice from hot water. The polymerization solvents were degassed and stored over activated 3 Å molecular sieves under an inert atmosphere, while the other solvents were ACS grade. 2,6-Bis(trimethyltin)-4,8-bis-ethylhexyloxy-benzo [1,2-*b*:4,5-*b′*]dithiophene (M1) was purchased from Sigma-Aldrich. Compounds 2-(6-bromohexyl)-4,7-bis(5-bromothiophen-2-yl)-2*H*-benzo[*d*][1,2,3]triazole [9] and 4,7-bis(5-bromothiophen-2-yl)-2-(2-octyldodecyl)-2*H*-benzo[*d*][1,2,3]triazole [43,54] (M2) were prepared according to previously reported methods. The synthesis of octyl 2-(3-hydroxy-6-oxo-6*H*-xanthen-9-yl)benzoate (FOE) and FOE-based monomer (M3) are given in ESI.

### 3.2. Measurements

The ^1^H and ^13^C NMR spectra were recorded on a Bruker AVANCE III HD 400 spectrometer (Bruker Corporation, Karlsruhe, Germany) in deuterated solvents. Chemical shifts were reported as δ values (ppm) relative to an internal tetramethylsilane (TMS) standard. Number-average (M_n_) and weight-average (M_w_) molecular weights were determined by size exclusion chromatography (SEC) at 25 °C on a Wyatt Technology Dawn EOS HPLC (Wyatt Technology, Santa Barbara, CA, USA) instrument equipped with a Knauer pump, three PLgel 5µMixed-C columns, and a static light-scattering (EA-02 Dawn Eos Enhanced Optical System) detector. The flow rate was 1.0 mL·min^−1^ using tetrahydrofuran (THF) as eluent. All samples were prepared at 1.0 mg·mL^−1^ in THF and were filtered through a 0.45 µm nylon filter. The calibration curve was produced with a series of monodisperse polystyrene standards. Thermogravimetric analysis (TGA) measurements were performed with a PerkinElmer Thermogravimetric Analyzer TGA 4000 (PerkinElmer, Waltham, MA, USA) from 25 °C to 800 °C at a heating rate of 20 °C·min^−1^. Differential Scanning Calorimetry (DSC) analysis of samples was performed in a PerkinElmer DSC 4000 from 25 °C to 300 °C at 20 °C·min^−1^ heating and cooling rates. TGA and DSC measurements were carried out under N_2_ atmosphere at 20 mL·min^−1^. UV-vis absorption spectra were collected in a Hewlett Packard 8453 spectrophotometer (Hewlett Packard, Palo Alto, CA, USA) using 1 cm path length quartz cells. For solid-state measurements, polymer solutions were spin-coated onto glass plates. Fluorescence spectra were recorded with an LS55 PerkinElmer fluorescence spectrometer using a 2.5 nm width slit for emission. The samples were excited at the wavelength of maximum absorption of the polymers in the solution. Fluorescence lifetimes were determined in a LifeSpecII fluorescence lifetime spectrometer (Edinburgh Instruments, Livingston, UK). The samples were excited with 458 nm and 506 nm laser diodes and the emission was collected at 558 nm for FOE, 574 nm for P1, and 611 nm for P2. The emission decays were analyzed using previously published procedures [55,56]. The quality of the fits was established using the chi-square (χ^2^) statistic, and the IRF was obtained using Ludox scattering solutions. Cyclic voltammetry (CV) experiments were conducted on a BASI^®^ EC Epsilon Potentiostat/Galvanostat (EClipse™, West Lafayette, IN, USA) at a scan rate of 100 mV·s^−1^ using a platinum disk as working electrode, Ag/AgCl (3M) as reference electrode and Pt wire as counter electrode in an anhydrous and argon-saturated solution of 0.01 M of tetrabutylammonium hexafluorophosphate (Bu_4_NPF_6_) in dried acetonitrile. A flow of high-purity argon was maintained during the measurements. The working electrode was modified by drop-casting solutions of polymers in chloroform (2 mg·mL^−1^). Under these experimental conditions, the oxidation potential (E_ox_) of ferrocene was 0.315 V versus Ag/AgCl and 0.447 V versus SCE. The HOMO and LUMO energy levels were determined from oxidation and reduction onset from the voltammogram profiles. The absolute potential of the SCE electrode is −4.7 eV in vacuum.

### 3.3. OPV Devices Fabrication

The OPV device architecture was as follows: ITO|MoO_x_|polymer:PC_61_BM|Bphen|Liq|Al. 1.5 × 1.5 inch glass substrates with a 1000 Å pre-patterned indium tin oxide (ITO) layer (Tinwell Technology Ltd., Hong Kong, China) were used for device fabrication. The substrates showed ~15 Ω·sq^−1^ surface resistance and ~90% optical transparency over the solar spectrum. The cleaning procedure of the substrates was conducted inside a laminar flow hood and included scrubbing with detergent solution, rinsing with DI water, ultrasonic baths for 10 min of DI water, acetone, and isopropanol, and lastly, flushing with nitrogen gas to dry the substrates. All films based on small-molecules were deposited by thermal evaporation in a vacuum chamber (<5.0·10^−6^ Torr). An 8 nm layer of molybdenum oxide (MoO_x_) was deposited at about 0.3 Å·s^−1^ on ITO as the hole-injecting layer (HIL). Subsequently, the samples were removed from the evaporator coater, and 100 µL of 10 mg·mL^−1^ P1/P2:PC_61_BM (1:1 weight ratio) in a chlorobenzene:1,8-diiodooctane (DIO; 99.5:0.5 *v*/*v*) solution was spin-coated at 2000 r.p.m. for 60 s over each substrate (ITO|MoO_x_) to generate a film thickness of 55–65 nm. Before use, the donor-acceptor blend was stirred at 60 °C for 24 h. Then, 3% *v*/*v* of DIO was added to the mixture and stirred for 1 h. The mixture was filtered through a 0.45 µm nylon filter prior to spin-coating. The devices were loaded back into the vacuum chamber to deposit 8 nm of 4,7-diphenyl-1,10-phenanthroline (Bphen) as the electron transport layer (ETL) and 1 nm of 8-hydroxyquinolinato lithium (Liq) as the electron-injecting layer (EIL). The deposition rates for Bphen and Liq were 4.0 Å·s^−1^ and 1.0·Å s^−1^, respectively. Finally, the devices were completed with a 1000 Å top aluminum electrode, deposited at a constant rate of 5.0 Å·s^−1^. Four identical OPV devices with an active area of 0.1 cm^2^ were produced on a single substrate. The thickness of the films was measured with a Profilm3D optical profilometer (Filmetrics, Unterhaching, Germany). The current density–voltage (J-V) characteristics were recorded with a Keithley 2400 source meter (Keithley Instruments, Inc., Cleveland, OH, USA) in the dark and under irradiation at 80 mW·cm^−2^ generated by a Solux 3SS4736 with a 50W 47K halogen lamp. The intensity was calibrated with a Hamamatsu S1787-12 silicon photodiode. External quantum efficiency (EQE) measurements were performed with a SpectraPro 275 monochromator (Action Research Corp., Acton, Massachusetts, USA). The hole mobility µ_h_ of the active layer was measured using the space charge limited current (SCLC) model ($J=\frac{9}{8}\varepsilon_{r}\varepsilon_{0}\mu_{h}\frac{V^{2}}{L^{3}}$), where J corresponds to the dark density current (mA·cm^−2^), $\varepsilon_{r}$ to the relative dielectric constant of the transportation medium (usually 3 for conjugated polymers), $\varepsilon_{0}$ to the permittivity of free space (8.854·10^−13^ mAs·V^−1^cm^−1^), $\mu_{h}$ to the hole mobility (cm^2^·V^−1^s^−1^), and V to the internal voltage (V = V_appl_ − V_bi_), where V_appl_ is the applied voltage, V_bi_ is the built-in potential, and L is the thickness of the active layer [57]. Hole-only devices were prepared by replacing the Liq layer with 8 nm MoO_x_ as an electron-blocking layer between Bphen and the cathode.

### 3.4. General Procedure for Polymerizations

A 25 mL oven-dry two-neck round-bottom flask was charged with the monomers (copolymer = 0.15 mmol of M1 and M2. Terpolymer = 0.15 mmol of M1 and 0.075 mmol of M2 and M3), Pd_2_(dba)_3_ (2 mol%) and P(*o*-tol)_3_ (8 mol%) and was then connected to a condenser. The flask and condenser were capped with septa and purged through vacuum/nitrogen filling (3 times). Then, anhydrous, oxygen-free toluene (7.5 mL, 0.02 M) was added via a syringe. The reaction mixture was vigorously stirred in an oil bath, from room temperature to 110 °C for 30 min under a nitrogen atmosphere. The reaction was removed from the oil bath, and 5 mL of *o*-dichlorobenzene were added to the reaction mixture. After cooling at room temperature, the mixture was precipitated in methanol, filtered through a 0.45 μm nylon filter, and washed by continuous Soxhlet extraction using methanol, *n*-hexane, and chloroform. The chloroform fraction was reduced to 5–10 mL and slowly poured in methanol. The precipitate was filtered through a 0.45 μm nylon filter and the polymer was then dried at 50 °C for 24 h. Copolymer P1 was obtained with an 85% yield, while terpolymer P2 had a 69% yield.

## 4. Conclusions

In this work, we synthesized a π-conjugated terpolymer (P2) based on BTz and BDT with a fluorescein derivative (FOE) attached to its backbone to promote the FRET phenomenon between them and used it as photoactive material in organic OSC. In addition, an alternating CP (P1) with an analogous structure was synthesized for comparison. Both polymers exhibited good thermal stability up to 360 °C, with optical bandgaps of about 2.0 eV. The LUMO and HOMO energy levels were −3.51 eV and −5.48 eV for P1 and −3.28 eV and −5.30 eV for P2. The significant overlap between FOE emission and P2 absorption was studied with time-resolved fluorescence suggesting the occurrence of the FRET process. To evaluate the effect of the FRET on P2-based OSCs, bulk-heterojunction devices with PC_61_BM as an acceptor were fabricated. Preliminary results showed poor device performance, probably due to un-optimized morphological structures and the formation of hole trap states between P2 and FOE. Nevertheless, improved photovoltaic parameters for P2 compared to P1 were observed due to the FRET process. These findings are promising for the development of improved and low-cost CPs.

## Figures and Tables

**Table 1:** Thermal, optical, and electrochemical properties of P1 and P2.

Sample	TDT_10%_ (°C) ^1^	λ_max_^sol^ (nm) ^2^	λ_max_^film^ (nm) ^2^	λ_max_^em^ (nm) ^3^	$E_{g}^{opt}$ Sol (eV) ^4^	$E_{g}^{opt}$ Film (eV) ^4^	E_HOMO_ (eV) ^5^	E_LUMO_ (eV) ^5^
P1	364	524	535/574	610	2.04	1.97	−5.48	−2.75 (−3.51) ^6^
P2	379	499	501	575	2.08	2.02	−5.30	−3.31 (−3.28) ^6^
FOE	-	460/490	-	556	2.37	-	−5.28	−4.00

^1^ Temperature at 10% weight loss. ^2^ Absorption maximum taken from UV-vis spectra of the polymer in chloroform dilute solution or solid-state. ^3^ Emission maximum from fluorescence spectra. ^4^ Calculated from the absorption edge of the polymer in solution/thin-film: $E_{g}^{\mathrm{opt}}$ = 1240·λ_edge_^−1^ eV. ^5^ E_HOMO_ = −e($E_{\mathrm{on}}^{\mathrm{ox}}\;$ + 4.4) eV/E_LUMO_ = −e($E_{\mathrm{on}}^{\mathrm{red}}$ + 4.4) eV. ^6^ E_LUMO_ = E_HOMO_ + $E_{g}^{\mathrm{opt}}$ film.

**Table 2:** Fluorescence lifetimes for P1, P2, and FOE.

Sample	λ_exc_/λ_em_ (nm)	τ_1_ (A1) (ns)	τ_2_ (A2) (ns)	τ_3_ (A3) (ns)	χ2
P1	458/574	0.22 (43%)	0.90 (57%)	-	1.088
P1	506/574	0.12 (44%)	0.84 (56%)	-	1.055
P2	458/611	0.06 (50%)	0.87 (35%)	1.82 (15%)	1.126
P2	506/611	-	0.81 (67%)	1.75 (33%)	1.159
FOE	458/558	-	-	2.64 (100%)	0.953

**Table 3:** Photovoltaic parameters and efficiencies of BHJ solar cell devices.

Sample	V_oc_ (V)	J_sc_ (mA·cm^−2^)	J_sc_ (EQE) (mA·cm^−2^)	FF	PCE (%)	µ_h_ (cm^2^·Vs^−1^)
P1	0.26 ± 0.02	0.11 ± 0.03	-	0.25 ± 0.02	0.01 ± 0.00	-
P2	0.55 ± 0.00	1.90 ± 0.08	1.94 ± 0.09	0.31 ± 0.00	0.41 ± 0.02	3.3 × 10^−4^ ± 3.9 × 10^−5^

## Data Availability

The data presented in this study are available on request from the corresponding authors.

## Supplementary Materials

The following supporting information can be downloaded at: https://www.mdpi.com/article/10.3390/ijms232112901/s1.
