# Supplementary material for: New Benzotriazole and Benzodithiophene-Based Conjugated Terpolymer Bearing a Fluorescein Derivative as Side-Group: In-Ternal Förster Resonance Energy Transfer to Improve Organic Solar Cells
Source: Int J Mol Sci. 2022 Oct 26;23(21):12901. doi: 10.3390/ijms232112901 (PMC9657233; doi:10.3390/ijms232112901)
Supplement: Supplementary file 1 [file ijms-23-12901-s001.zip › ijms-1986328-SI.pdf]

Synthesis and characterization of precursors and monomers

Monomer 2,6-bis(trimethyltin)-4,8-bis-ethylhexyloxy-benzo[1,2-*b*:4,5-*b′*]dithiophene (M1) was commercially available, while compounds 2-(6-bromohexyl)-4,7-bis(5-bromothiophen-2-yl)-2*H*-benzo[*d*][1,2,3]triazole (^{1}H NMR (400 MHz, $CDCl_3$) δ 7.76 (d, *J* = 3.9 Hz, 2H), 7.48 (s, 2H), 7.12 (d, *J* = 3.9 Hz, 2H), 4.80 (t, *J* = 7.1 Hz, 2H), 3.40 (t, *J* = 6.7 Hz, 2H), 2.26 – 2.13 (m, 2H), 1.91 – 1.82 (m, 2H), 1.61 – 1.51 (m, 2H), 1.50 – 1.39 (m, 2H); ^{13}C NMR (101 MHz, $CDCl_3$) δ 141.87, 141.31, 131.02, 127.07, 123.12, 122.40, 113.37, 56.79, 33.74, 32.62, 29.92, 27.68, 25.88) [1] and 4,7-bis(5-bromothiophen-2-yl)-2-(2-octyldodecyl)-2*H*-benzo[*d*][1,2,3]triazole (M2; ^{1}H NMR (400 MHz, $CDCl_3$) δ 7.77 (d, *J* = 3.8 Hz, 2H), 7.50 (s, 2H), 7.11 (d, *J* = 3.8 Hz, 2H), 4.72 (d, *J* = 6.5 Hz, 2H), 2.32-2.23 (m, 1H), 1.36-1.20 (m, 32H), 0.91-0.85 (m, 6H); ^{13}C NMR (101 MHz, $CDCl_3$) δ 141.73, 141.43, 130.96, 126.98, 123.08, 122.17, 113.32, 60.13, 39.27, 32.08, 32.04, 31.63, 30.05, 29.82, 29.79, 29.73, 29.51, 29.47, 26.42, 22.84, 22.82, 14.27) [2, 3] were prepared according to previously reported methods. Synthesis of octyl 2-(3-hydroxy-6-oxo-6*H*-xanthen-9-yl)benzoate (FOE) and Octyl 2-(3-(6-(4,7-bis(5-bromothiophen-2-yl)-2*H*-benzo[*d*][1,2,3]triazol-2-yl)hexyloxy)-6-oxo-6*H*-xanthen-9-yl)benzoate (M3) are described herein (Scheme S1).

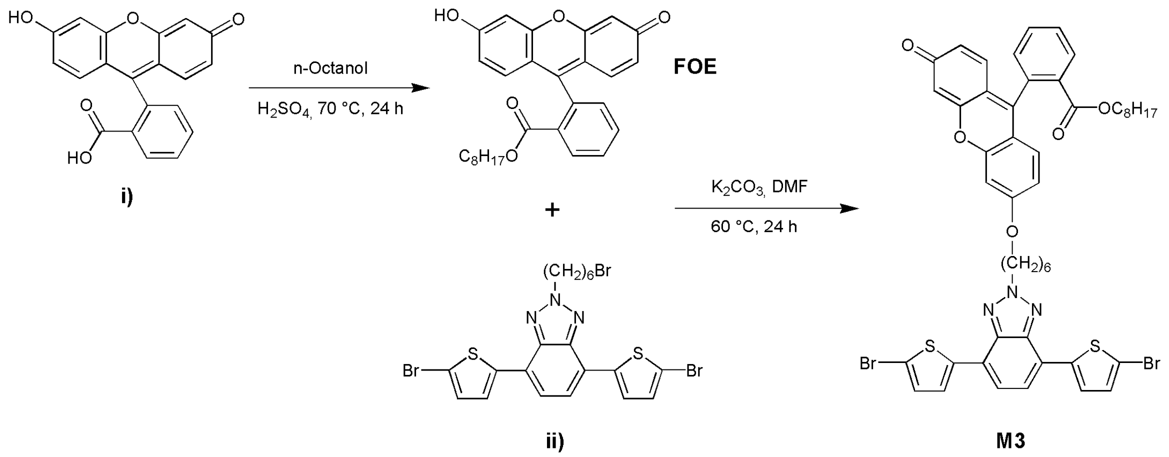

Scheme S1. Synthetic routes to FOE and M3.

Octyl 2-(3-hydroxy-6-oxo-6H-xanthen-9-yl)benzoate (FOE). FOE was prepared from the modification of previously reported procedures [4,5]. Briefly, Fluorescein (**i**; 1.00 g) was dissolved in *n*-octanol (3 mL) into a 10 mL microwave vial. The vial was sealed and H_2SO_4 (0.5 mL) was then added dropwise via syringe. The mixture was heated for 24 h at 70 °C. The mixture was cooled to room temperature and poured into diethyl ether. The precipitated was filtered through a nylon filter and then dissolved in a 1:1 v/v ethanol/water solution at 75 °C. The pH of the solution was adjusted to 5 using a 5 % w/v Na_2CO_3 solution to promote FOE precipitation. The precipitate was filtered through a 0.45 μm nylon filter and vacuum-dried to give an orange solid (yield: 89 %). ^{1}H NMR (400 MHz, $CDCl_3$) δ 8.98 (s, 1H), 8.25 (d, *J* = 7.7 Hz, 1H), 7.69 (dd, *J* = 11.8, 7.4 Hz, 2H), 7.29 (d, *J* = 7.2 Hz, 1H), 6.98 (d, *J* = 9.2 Hz, 2H), 6.89 (s, 2H), 6.82 (d, *J* = 9.1 Hz, 2H), 3.92 (t, *J* = 6.5 Hz, 2H), 1.30 – 1.19 (m, 4H), 1.13 (s, 6H), 1.03 – 0.94 (m, 2H), 0.82 (t, *J* = 7.0 Hz, 3H). ^{13}C NMR (101 MHz, $CDCl_3$) δ 175.80, 165.51, 157.99, 156.19, 134.17, 132.54, 131.36, 130.75, 130.71, 130.38, 129.92, 122.15, 114.89, 103.83, 65.93, 31.81, 29.17, 29.10, 28.27, 25.88, 22.68, 14.18.

Octyl 2-(3-(6-(4,7-bis(5-bromothiophen-2-yl)-2H-benzo[d][1,2,3]triazol-2-yl)hexyloxy)-6-oxo-6H-xanthen-9-yl)benzoate (M3). A mixture of FOE (0.22 g, 0.50 mmol), K_2CO_3 (0.21 g, 1.50 mmol), and DMF (6 mL) was heated to 60 °C for 30 min. 2-(6-bromohexyl)-4,7-bis(5-bromothiophen-2-yl)-2*H*-benzo[*d*][1,2,3]triazole (**ii**; 0.30 g, 0.50 mmol) was added portionwise to the mixture and the solution was stirred for 24 h at 60 °C. Then, water was added and the reaction mixture was extracted with ethyl acetate. The combined organic layers were dried over anhydrous Na_2SO_4 and filtered. The solvent was removed *in vacuo* and the residue was purified by column chromatography on silica gel (*n*-hexane/dichloromethane, 3:2 v/v) to afford a red solid (0.43 g, 88%). ^{1}H NMR (400 MHz, $CDCl_3$) δ 8.25 (d, *J* = 7.2 Hz, 1H), 7.75 (d, *J* = 3.7 Hz, 2H), 7.69 (dd, *J* = 13.3, 7.7 Hz, 2H), 7.48 (s, 2H), 7.27 (d, *J* = 8.6 Hz, 1H), 7.09 (d, *J* = 3.7 Hz, 2H), 6.85 (s, 3H), 6.67 (d, *J* = 8.5 Hz, 1H), 6.55 (d, *J* = 9.3 Hz, 1H), 6.45 (s, 1H), 4.82 (t, *J* = 6.7 Hz, 2H), 4.02 (t, *J* = 6.0 Hz, 2H), 3.95 (s, 2H), 2.27 – 2.17 (m, 2H), 1.90 – 1.76 (m, 2H), 1.64 – 1.53 (m, 2H), 1.52 – 1.44 (m, 2H), 1.34 – 1.18 (m, 9H), 1.17 – 1.07 (m, 2H), 1.06 – 0.94 (m, 1H), 0.85 (t, *J* = 7.0 Hz, 3H). ^{13}C NMR (101 MHz, $CDCl_3$) δ 185.75, 165.68, 159.05, 154.40, 141.85, 141.26, 134.29, 132.61, 131.42, 130.97, 130.58, 130.42, 129.75, 126.97, 123.08, 122.38, 114.92, 113.40, 105.85, 68.72, 65.89, 32.04, 31.87, 29.82, 29.22, 29.14, 28.33, 26.32, 25.91, 22.73, 14.22.

Synthesis of polymers

P1 and P2 were synthesized by Stille polycondensation using $Pd_2(dba)_3$/P(*o*-tol)$_3$ as a catalytic system at 110 °C in toluene (Scheme S2).

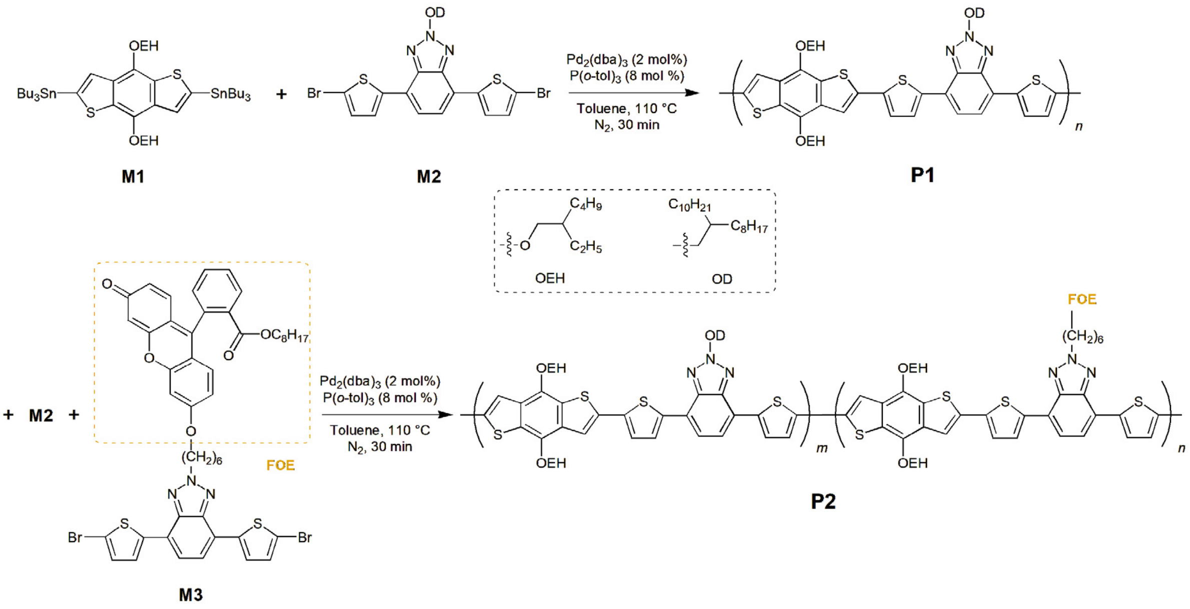

Scheme S2. Synthetic route to P1 and P2.

P1: M1 (0.116 g, 0.15 mmol), M2 (0.108 mg, 0.15 mmol), $Pd_2(dba)_3$ (2.7 mg, 2% mol), P(*o*-tol)$_3$ (3.7 mg, 8% mol), and anhydrous toluene (7.50 mL, 0.02 M). Yield = 85 %. ^{1}H NMR in Figure S1.

P2: M1 (0.116 g, 0.15 mmol), M2 (0.054 g, 0.075 mmol), M3 (0.073 g, 0.075 mmol), $Pd_2(dba)_3$ (2.7 mg, 2% mol), P(*o*-tol)$_3$ (3.7 mg, 8% mol), and anhydrous toluene (7.50 mL, 0.02 M). Yield = 69 %. ^{1}H NMR in Figure S2.

^{1}H NMR characterization

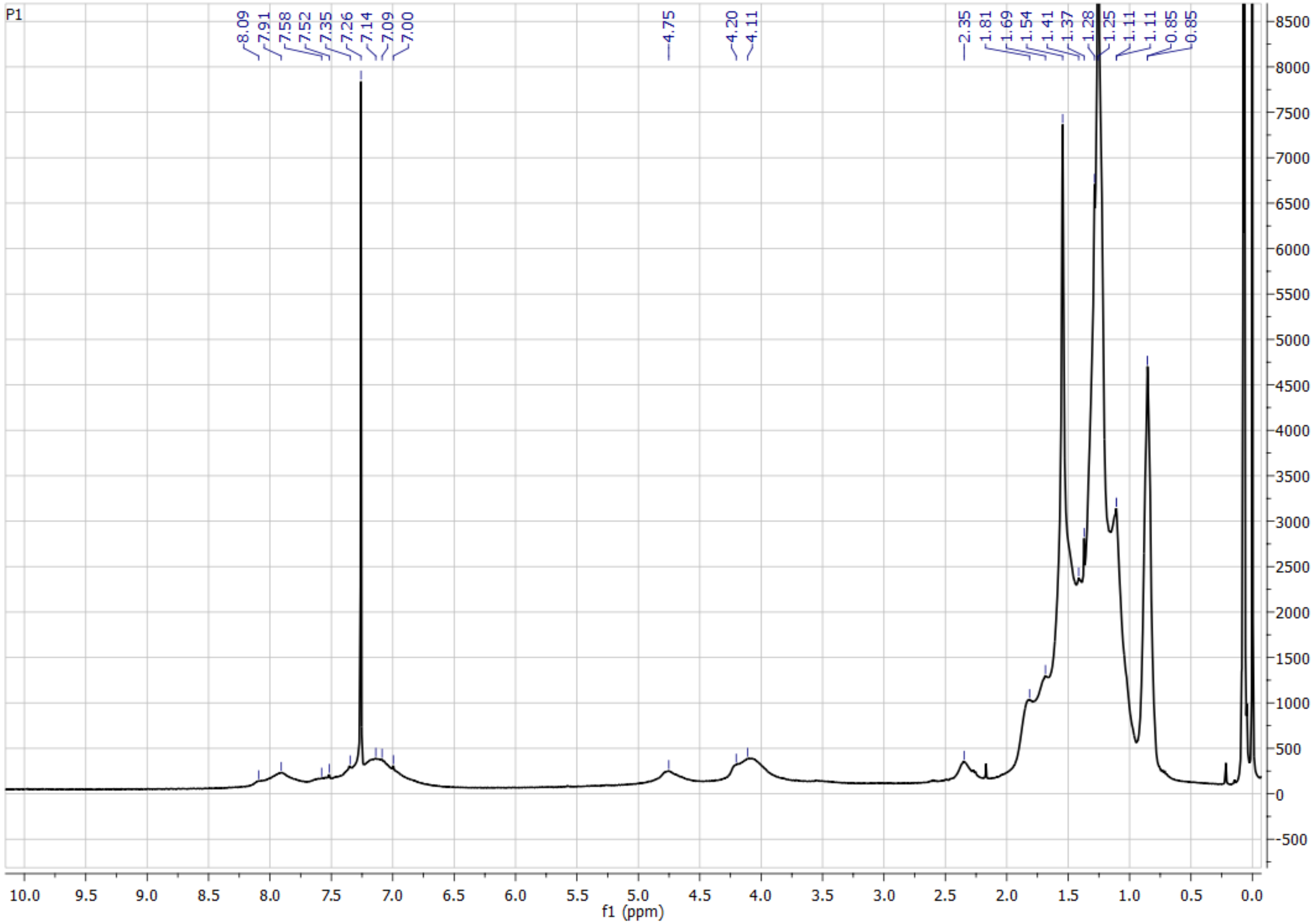

Figure S1. ^{1}H NMR spectra of P1 in $CDCl_3$.

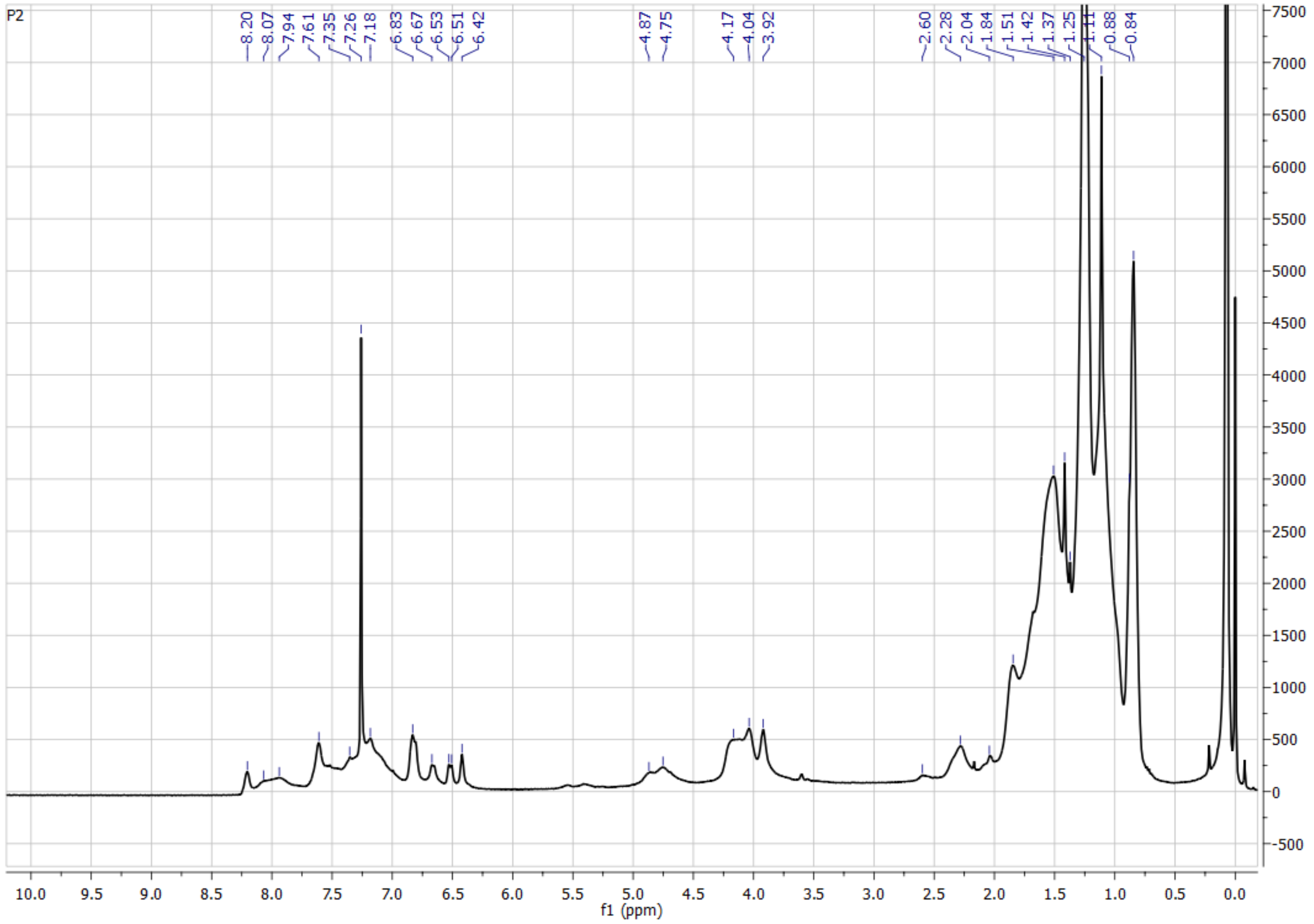

Figure S2. ^{1}H NMR spectra of P2 in $CDCl_3$.

Thermal characterization

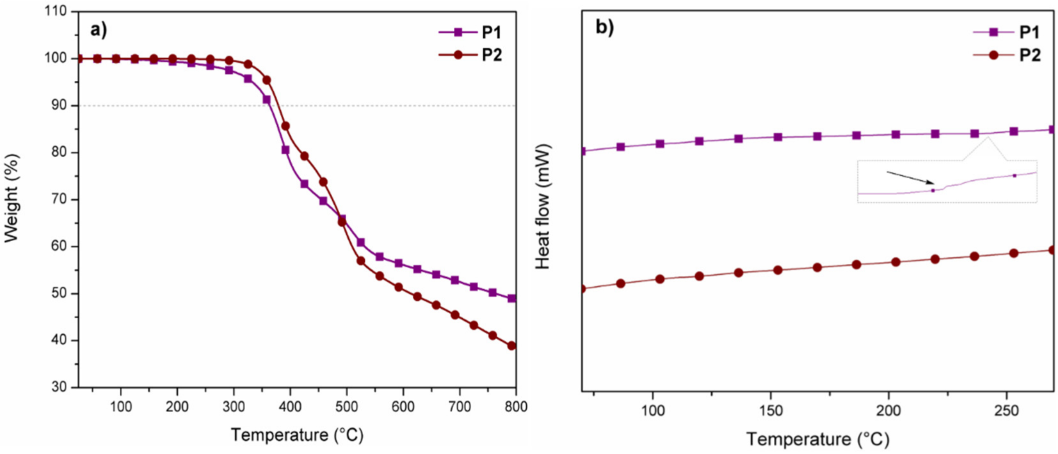

Figure S3. (**a**) TGA thermograms and (**b**) DSC traces for second heating for P1-P2.

References

1. Jessop, I. A.; Bustos, M.; Hidalgo, D.; Terraza, C. A.; Tundidor-Camba, A.; Pardo, M. A.; Fuentealba, D.; Hssein, M.; Bernede, J. C., Synthesis of 2H-benzotriazole based donor-acceptor polymers bearing carbazole derivative as pendant groups: Optical, electronical and photovoltaic properties. International Journal of Electrochemical Science 2016, 9822-9838.
2. Cevher, S. C.; Unlu, N. A.; Ozelcaglayan, A. C.; Apaydin, D. H.; Udum, Y. A.; Toppare, L.; Cirpan, A., Fused structures in the polymer backbone to investigate the photovoltaic and electrochromic properties of donor-acceptor-type conjugated polymers. Journal of Polymer Science Part A: Polymer Chemistry 2013, 51 (9), 1933-1941.
3. Zhang, Z.; Peng, B.; Liu, B.; Pan, C.; Li, Y.; He, Y.; Zhou, K.; Zou, Y., Copolymers from benzodithiophene and benzotriazole: synthesis and photovoltaic applications. Polymer Chemistry 2010, 1 (9).
4. Rokitskaya, T. I.; Sumbatyan, N. V.; Tashlitsky, V. N.; Korshunova, G. A.; Antonenko, Y. N.; Skulachev, V. P., Mitochondria-targeted penetrating cations as carriers of hydrophobic anions through lipid membranes. Biochimica et Biophysica Acta (BBA) - Biomembranes 2010, 1798 (9), 1698-1706.
5. Brown, L.; Halling, P. J.; Johnston, G. A.; Suckling, C. J.; Valivety, R. H., The synthesis of some water insoluble dyes for the measurement of pH in water immiscible solvents. Journal of the Chemical Society, Perkin Transactions 1 1990, (12), 3349-3353.
